# Transcriptome Profiling Analysis Reveals the Potential Mechanisms of Three Bioactive Ingredients of Fufang E’jiao Jiang During Chemotherapy-Induced Myelosuppression in Mice

**DOI:** 10.3389/fphar.2018.00616

**Published:** 2018-06-13

**Authors:** Xue Li, Yan Zhang, Zhuping Hong, Shuqing Gong, Wei Liu, Xiangshan Zhou, Yangen Sun, Jing Qian, Haibin Qu

**Affiliations:** ^1^Pharmaceutical Informatics Institute, College of Pharmaceutical Sciences, Zhejiang University, Hangzhou, China; ^2^National Engineering Research Center for Gelatin-based Traditional Chinese Medicine, Dong-E-E-Jiao Co., Ltd., Liaocheng, China

**Keywords:** martynoside, R-notoginsenoside R2, 20S-ginsenoside Rg2, hematopoietic, myelosuppression, RNA-seq

## Abstract

Although multiple bioactive components have been identified in Fufang E’jiao Jiang (FEJ), their hematopoietic effects and molecular mode of action *in vivo* are still not fully understood. In the current study, we analyzed the effects of martynoside, R-notoginsenoside R2 (R2), and 20S-ginsenoside Rg2 (Rg2) in a 5-fluorouracil-induced myelosuppression mouse model. Bone marrow nucleated cells (BMNCs) counts, hematopoietic progenitor cell colony-forming unit (CFU) assay, as well as flow cytometry analysis of Lin^-^/c-kit^+^/Sca-1^+^ hematopoietic stem cell (HSC) population were conducted, and bone marrow cells were subjected to RNA sequencing. The transcriptome data were processed based on the differentially expressed genes. The results of the analysis show that each of the three compounds stimulates BMNCs and HSC growth, as well as burst-forming unit-erythroid and colony-forming unit granulocyte-monocyte colony expansion. The most relevant transcriptional changes appeared to be involved in regulation of hematopoietic cell lineage, NF-κB and TNF-α signaling, inhibition of inflammation, and acceleration of hematopoietic cell recovery. Notably, the individual compounds shared similar but specified transcriptome profiles. Taken together, the hematopoietic effects for the three tested compounds of FEJ are confirmed in this myelosuppression mouse model. The transcriptome maps of these effects provide valuable information concerning their underlying mechanisms and provide a framework for the continued study of the complex mode of action of FEJ.

## Introduction

Fufang E’jiao Jiang (FEJ) is an ancient “bu qi yang xue” formula used to benefit an individual’s qi and nourish blood and has been recognized as a medication for weakness and anemia from more than 400 years. In modern medicine, FEJ is widely used as a supportive reagent for chemotherapy-induced myelosuppression in East Asian countries, including China, Japan, and Korea. We previously reported that FEJ stimulates the hematopoiesis of bone marrow (BM) cells in mice with 5-fluorouracil (5FU)-induced myelosuppression (Shen et al., 2016). Unfortunately, as with other traditional Chinese medicines, there is only a limited amount of information on the beneficial effects and mechanism of FEJ.

FEJ is a multi-component formula that contains Asini Corii Colla, Ginseng Radix et Rhizoma Rubra, Rehmanniae Radix Praeparata, Codonopsis Radix, and Crataegi fructus (Liu et al., 2014). We recently utilized high-performance liquid chromatography–mass spectrometry (HPLC–MS), together with high-resolution mass spectrometry (HR-MS), to further characterize FEJ (Shen et al., 2016). In this previous analysis, 72 phytochemical constituents were identified, including the novel bioactive compounds martynoside, R-notoginsenoside R2 (designated R2), and 20S-ginsenoside Rg2 (designated Rg2).

**FIGURE 1:**
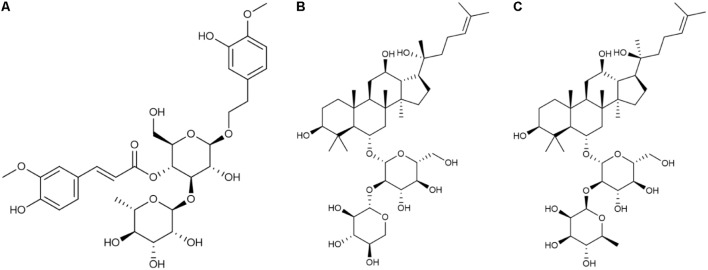
Chemical structure of the three compounds extracted from FEJ. **(A)** Martynoside. **(B)** R-notoginsenoside R2. **(C)** 20S-ginsenoside Rg2.

These three compounds have been shown to have numerous biological effects. Martynoside, identified as a component of Rehmanniae Radix Praeparata (Shen et al., 2016), is a plant phenylpropanoid glycoside (**Figure 1A**). It has several known pharmacological properties, including anti-sports anemia (Zhu et al., 2010) and retardation of skeletal muscle fatigue (Liao et al., 1999). Martynoside has also been shown to act as a novel natural selective estrogen receptor modulator (SERM) (Papoutsi et al., 2006). R2 has been identified as a component of Ginseng Radix et Rhizoma Rubra (**Figure 1B**) (Shen et al., 2016). According to a recent report, R2 has anti-apoptosis properties and acts as a neuroprotective agent against Parkinson’s disease (Meng et al., 2013). A panax notoginoside (PNS) containing R2, as well as other notoginosides, functions as an anti-inflammatory, in addition to inhibiting platelet aggregation, lengthening clotting time, reducing blood fat and blood pressure, and promoting hematopoietic cell (HPC) proliferation (Su et al., 2005; Gao et al., 2007). This PNS is widely used in treating cardiovascular and neurodegenerative diseases (Yang et al., 2014). Rg2 is a compound medicine that is also found in Ginseng Radix et Rhizoma Rubra (**Figure 1C**) (Shen et al., 2016) and is widely used to treat cardio-cerebrovascular diseases (Fu et al., 2015). It also has a number of other properties, including anti-diabetic activity (Yuan et al., 2012), anti-depressant-like activity (Ren et al., 2017), neuroprotective activity in Alzheimer’s disease (Cui et al., 2017), antioxidative activity following UV-B radiation injury (Kang et al., 2016), and anti-inflammatory activity in vascular disease (Cho et al., 2013). Although these three components of FEJ probably play a significant role in its beneficial effects, very little is known about their hematopoietic functioning.

In this study, we investigated the hematopoietic effects and underlying mechanisms of martynoside, R2, and Rg2 in 5FU-induced BM-depressed mice. We utilized RNA sequencing (RNA-seq) technologies to identify regulatory genes and the molecular pathways that modulate their function. The results of this analysis provide significant insight into the beneficial effects of FEJ in myelosuppression and provide a foundation for future analyses of this and other traditional Chinese medicines.

## Materials and Methods

### Chemicals

Martynoside (purity ≥95%, determined by HPLC), R2 (purity ≥95%, determined by HPLC), and Rg2 (purity ≥98%, determined by HPLC) were purchased from the Yuanye Medical Technology and Development Corporation (Shanghai, China). 5FU was obtained from Sigma-Aldrich (Santa Anta, CA, United States). Mouse methylcellulose medium containing recombinant cytokines (MethoCult medium) for use in the colony-forming unit (CFU) assays was purchased from Stem Cell Technologies (Vancouver, Canada). 7-AAD, APC Mouse Lineage Antibody Cocktail, FITC Rat Anti Mouse Ly-6A/E and their relevant isotype control antibodies were purchased from BD Biosciences (Franklin Lakes, NJ, United States). PE anti-mouse CD117 Antibody was purchased from Biolegend (San Diego, CA, United States). All the cell culture reagents were purchased from Invitrogen (San Diego, CA, United States), unless specified otherwise.

### Animals

BALB/c mice were purchased from the Shanghai Laboratory Animal Company (SLAC, Shanghai, China) and maintained in the following conditions: 24–26°C, 40–60% humidity, 12-h light/dark cycle, with food and water at will. All of the animal experiments were approved by the Animal Care and Use Committee of Zhejiang University and followed the *Guide for the Care and Use of Laboratory Animals* (Mason and Matthews, 2012).

### Administration of 5FU and Drugs

The mice were randomly divided into five groups: a control group (Control), a myelosuppression model group (Model), a martynoside treatment group (Martynoside), an R2 treatment group (R2), and an Rg2 treatment group (Rg2). In the model group, mice were intraperitoneally injected with 200 mg/kg 5FU (Wang et al., 2015). In the treatment groups, the specified compound was orally given to the mice at an approximate dosage of 20 mg/kg/day immediately after 5FU administration. The control and model group received an equivalent amount of distilled water correspondingly. On day 10 (*n* = 6 per group) or day 15 (*n* = 2–3 per group), the mice were euthanatized, and their BM cells were isolated for further analyses. Samples collected on both days were used for bone marrow nucleated cells (BMNCs) counting and CFU assay. In addition, flow cytometry (FCM) analysis of hematopoietic stem cells (HSC) population was performed with samples collected on day 10, and RNA-seq was performed with that collected on day15, respectively.

### Preparation and Counting of BMNCs

The mice’s BM cells were collected and counted as previously described (Wang et al., 2015). A 23-gauge needle was used to flush cells out from the medullary canal of the tibia and femoral bone, and a 100-μm cell strainer was used to obtain single-cell suspensions. After lysis of red blood cells, the BMNCs count and viability were determined by the trypan blue exclusion method, using the Countess^TM^ automated cell counter (Thermo Fisher Scientific, Waltham, MA, United States).

### Colony-Forming Unit Assay

The CFU assay was carried out following the manufacturer’s instructions. BMNCs were adjusted to 2 × 10^5^ cells/mL, and 0.3 mL of the cell suspension was added to 3 mL of MethoCult medium and violently vortexed. Duplicated 1.1 mL aliquots of the mixture were then dispensed into 35-mm culture dishes (Stem Cell Technologies) with a 16-gauge blunt-end needle (Stem Cell Technologies) and incubated in a humidified incubator at 37°C and 5% CO_2_. On the 9th to 11th day of culture, burst-forming unit-erythroid (BFU-E, early erythroid population) and colony-forming unit granulocyte-monocyte (CFU-GM) cells were identified and counted.

### Flow Cytometry Analysis

Bone marrow nucleated cells were adjusted to 1 × 10^6^ cells/100 μL and were stained with APC Mouse Lineage Antibody Cocktail (containing CD3e (145-2C11), CD11b (M1/70), CD45R/B220 (RA3-6B2), Ly-76 (TER-119), Ly6G, Ly-6C (RB6-8C5)), PE anti-mouse CD117 Antibody and FITC Rat Anti Mouse Ly-6A/E (Sca-1) in 4°C for 30 min. Another portion of cells were stained with relevant isotope control antibodies correspondingly. After washing with PBS, cells were stained with 7-AAD for 10 min to eliminate interference of dead cells and sorted by BD FACSCalibur Cytometer (Franklin Lakes, NJ, United States). Data were analyzed using FlowJo v10.0.7 software (Li et al., 2015). Cells characterized with Lin^-^/c-kit^+^/Sca-1^+^ were recognized as HSC.

### RNA Extraction, Library Preparation, and Sequencing

Bone marrow cells were sent to the Beijing Genomic Institution^1^ (BGI, Shenzhen, China) for mRNA preparation and RNA-seq. Library construction and sequencing were performed on a BGISEQ-500. Clean tags were mapped to the reference genome and genes available at the Mice Genome Annotation Project^2^, with a perfect match or one mismatch allowed. The original sequencing data were deposited in public database of NCBI BioProject with the project ID being PRJNA407862^3^.

### RNA-seq Data Analysis

Gene expression, calculated by the fragments per kilobase of transcript per million mapped reads (FPKM) method using an RESM quantification tool (Li and Dewey, 2011), was presented as the mean FPKM. Differentially expressed genes (DEG) were defined according to the absolute value of their log2 (fold change) ≥1 and divergence probability ≥0.8 using the NOISeq method (Tarazona et al., 2011). Gene ontology (GO), pathway annotation, and enrichment analyses were carried out using the Gene Ontology database^4^ (taking corrected *p*-value ≤ 0.05 as a threshold), the KEGG pathway database^5^ (taking *Q* value ≤ 0.05 as a threshold), and DAVID^6^. A Venn diagram was constructed to obtain co-expressed DEG between (among) samples using Venny 2.1.0^7^. Hierarchical clustering analysis, combined with heat map construction, was conducted to compare gene fold change levels and tendency using cluster analysis software. The biological significance of DEG was explored using STRING^8^, and network visualization was performed using Cytoscape 3.5.0.

### Real-Time Quantitative PCR

Total RNA was isolated from BM cells using an Ultrapure RNA kit (CWBIO, Beijing, China), following the procedures provided by the manufacturer, and was reverse-transcribed into cDNA with a HiFiScript cDNA Synthesis kit (CWBIO, Beijing, China). qPCR was performed on a CFX96^TM^ Touch system (Bio-Rad, Hercules, CA) using an UltraSYBR mixture (CWBIO). The primer sequences, which were designed and synthesized by Sangon Biotech (Shanghai, China), are listed in **Table 1**. β-Actin was amplified as an endogenous reference gene. The relative expression was calculated with the ΔΔC_T_ method and expressed as the fold change in comparison to the control.

**Table 1:** Primer sequences for RT-qPCR.

Targets	Forward primer (5′–3′)	Reverse primer (5′–3′)
β-*actin*	GTATCCTGACCCTGAAGTACC	TGAAGGTCTCAAACATGATCT
*EpoR*	CGCTTGGAAGACTTGGTGTG	CCTGGTGCAGGCTACATGAC
*Csf1*	TTGCCAAGGAGGTGTCAGAA	GGCAATCTGGCATGAAGTCTC
*TNF-α*	AATAACGCTGATTTGGTGA	ACCCGTAGGGCGATTACA
*Nfkbiz*	TCCAAGTCAGTGGCTCTCCA	TGTTGATGTTGCTGCTGTGG
*Cxcl1*	ACCCAAACCGAAGTCATA	GGTGCCATCAGAGCAGT
*Tubb1*	CCTGCTTCTGCATCGACAAC	GTGATTCCGCTCATGGTCAA
*Acod1*	CCTGCTGACCACATCGAGAG	GCACAGGCCACATACTGGAA
*Mc5r*	CATCATGTGCAACTCCGTGA	GCCAAGGAGCCTACAAGGTC
*Fosl1*	TGAACCGGAAGCACTGCATA	GGAGGAGCAAGGTTCTGGTG
*Lif*	TGGTGGAGCTGTATCGGATG	AGCATTGAGCTTGACCTGGAG


### Statistical Analysis

Data are represented by their means ± standard errors of the means (SEM). Significant differences between groups were detected by multi-factor analysis of variance (one-way ANOVA) and *t*-test using GraphPad Prism software. A *p*-value less than 0.05 indicated statistical significance.

## Results

### Effects of Martynoside, R2, and Rg2 on BMNCs Counting and CFU Colony Numbers in 5FU-Induced Myelosuppression Mice

**FIGURE 2:**
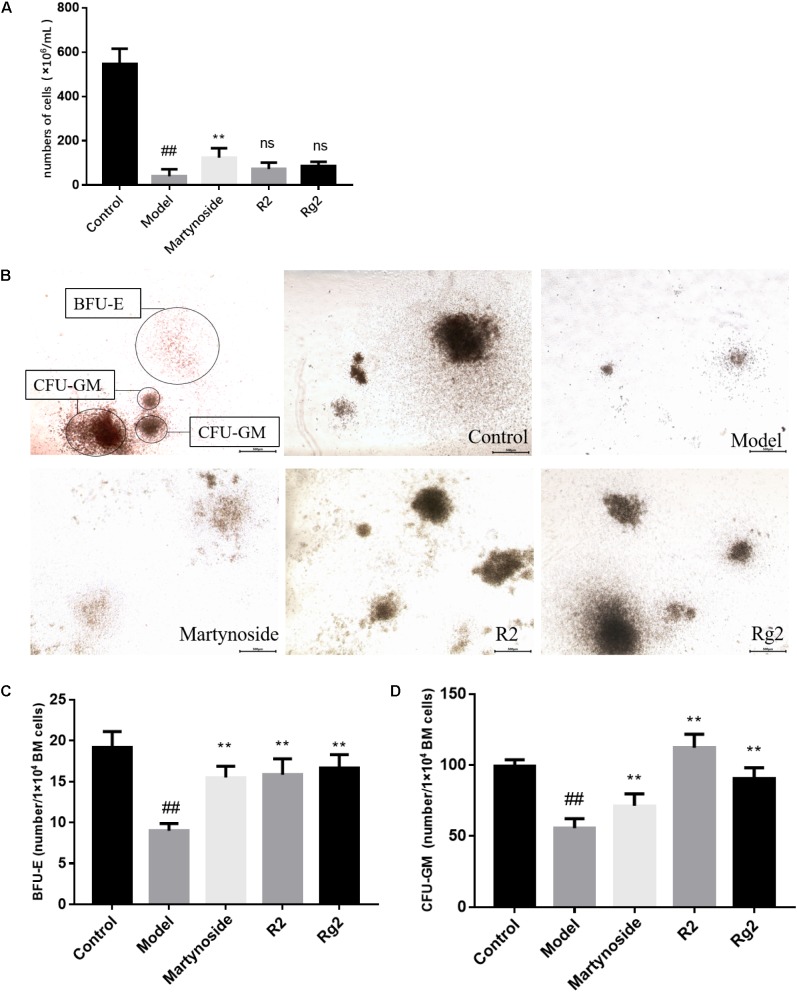
Effects of martynoside, R2, and Rg2 on BMNCs counting and CFU colony numbers in 5FU-induced myelosuppression mice. **(A)** The number of BMNCs from each group. Cells were isolated from each group and counted as described in Section “Materials and Methods.” The results are expressed as the means ± SEM (*n* = 5 for R2, *n* = 6 for other groups). **(B)** Representative image of colony formation in each treatment group. CFU-GM, colony-forming units granulocyte-monocyte; BFU-E, burst-forming unit-erythroid. **(C)** Number of BFU-E cells in each treatment group (*n* = 3). **(D)** Number of CFU-GM cells in each treatment group (*n* = 3). The results are expressed as the means ± SEM. ^##^*p* < 0.01, compared with the Control group; ^∗∗^*p* < 0.01, ns, no significant difference compared with the Model group.

In a previous study, we showed that three compounds from FEJ (i.e., martynoside, Rg2, and R2) promoted the proliferation of both mesenchymal stem cells (MSCs) and suspended HSCs that were extracted from 5FU-treated mice (Shen et al., 2016). In the present study, we assessed their effects on hematopoietic function on a myelosuppression mouse model *in vivo*. As shown in **Figure 2A**, on day 10, the number of BMNCs in the Model group was significantly less than that in the Control group (39.17 ± 31.56 vs. 546.67 ± 68.31, *p* < 0.05). Furthermore, in comparison to the Model group, martynoside treatment group had significantly more BMNCs (123.67 ± 42.66 vs. 39.17 ± 31.56, *p* < 0.05). No significant differences in the number of BMNCs were observed between the R2, Rg2, and Model groups (60.33 ± 39.22 and 85.33 ± 19.70, respectively, vs. 212 ± 39.59, *p* > 0.05).

The proliferation and differentiation patterns of BMNCs from the five treatment groups were assessed by CFU assay. After culturing in MethoCult medium, the CFUs were classified and enumerated *in situ* under a light microscope. As shown in **Figures 2B–D**, on day 10, in comparison to the Control group, the Model group had significant fewer BFU-E (19.17 ± 1.94 vs. 9.00 ± 0.89, *p* < 0.05) cells and significantly more CFU-GM cells (99.17 ± 4.17 vs. 55.67 ± 6.68, *p* < 0.05). In comparison to the Model group, treatment with martynoside, R2, and Rg2 resulted in an increase in the number of BFU-E and CFU-GM cells (BFU-E, 15.50 ± 1.38, 15.83 ± 1.94, and 16.67 ± 1.63, respectively, vs. 9.00 ± 0.89; CFU-GM, 71.33 ± 8.48, 112.33 ± 9.48, and 90.67 ± 7.55 vs. 55.67 ± 6.68, *p* < 0.05).

### Effects of Martynoside, R2, and Rg2 on HSC Population in 5FU-Induced Myelosuppression Mice

**FIGURE 3:**
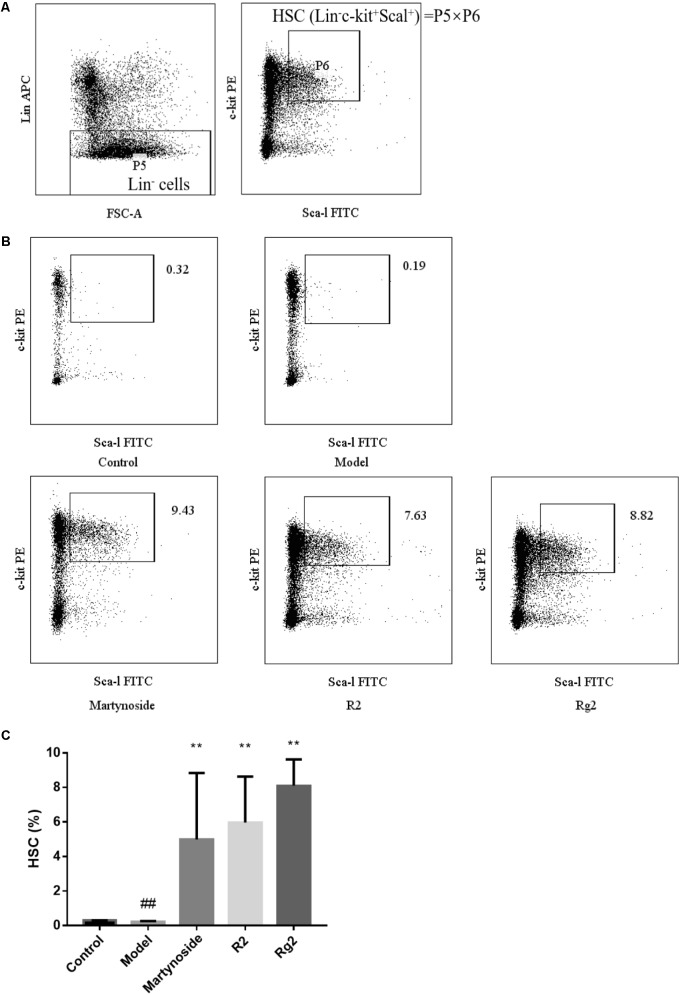
Effects of martynoside, R2, and Rg2 on HSC population in a 5FU-induced myelosuppression mouse model. **(A)** Gating strategy of HSC. P5 representing Lin^-^ population is further gated for c-kit^+^ and Sca-1^+^ population (P6). The HSC is defined as Lin^-^/c-kit^+^/Sca-1^+^ and the population presented is calculated from value of P5 × P6. **(B)** Representative image of flow cytometry in each treatment group. **(C)** HSC in each treatment group. The results are expressed as the means ± SEM (*n* = 5 for R2, *n* = 6 for other groups). ^##^*p* < 0.01, compared with the Control group; ^∗∗^*p* < 0.01, compared with the Model group.

On day 10 after 5FU treatment, HSC (Lin^-^c-kit^+^Sca-1^+^) in BM was analyzed. As shown in **Figure 3**, HSC in the Model group was significantly less than that in the control group (0.22 ± 0.04 vs. 0.30 ± 0.01, *p* < 0.01). Furthermore, in comparison to the Model group, martynoside, R2, and Rg2 treatment significantly increased HSC in BM (4.99 ± 3.85, 5.97 ± 2.66, 8.11 ± 1.52, respectively, vs. 0.22 ± 0.04, *p* < 0.01).

### Identification of DEG in BM Cells via RNA-seq

Next, on day 15, we performed an RNA-seq-based genome-wide gene expression study of BM cells to better understand the molecular changes involved in the observed treatment-mediated effects. After filtering low quality reads, an average of 24 million clean reads were obtained and 94.91% clean reads were mapped to the mice genome. The detailed summary of sequencing data for each sample was listed in **Supplementary Table S1**. A total of 17,759 genes in the Control group, 17,369 genes in the Model group, 17,302 genes in the Martynoside group, 17,245 genes in the R2 group, and 17,377 genes in the Rg2 treatment group were used for subsequent analyses.

**FIGURE 4:**
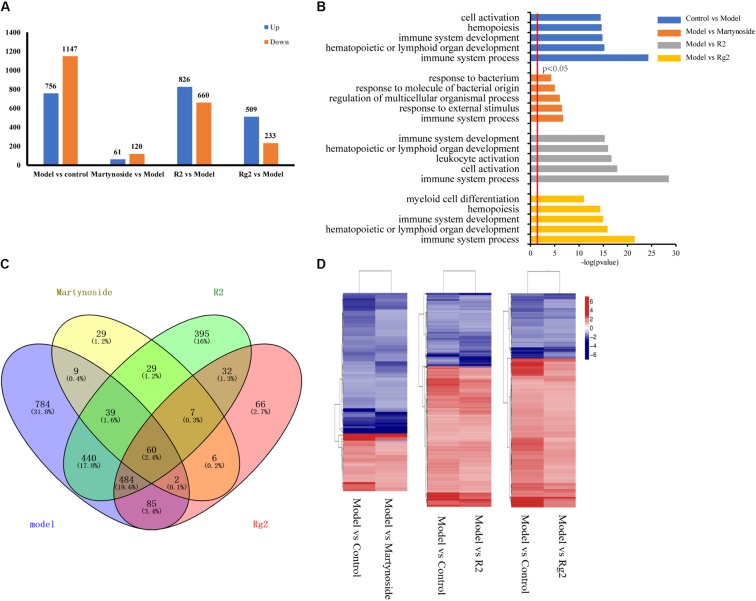
Gene expression profiling by RNA-seq. **(A)** Number of upregulated and downregulated genes in Model and each treatment group. **(B)** List of enriched GO term Biological Process for Model and each treatment group. **(C)** Venn diagram of DEGs between Control vs. Model (model), Martynoside vs. Model (Martynoside), R2 vs. Model (R2), and Rg2 vs. Model (Rg2). **(D)** Heat map of common DEGs.

We first analyzed the DEG and their GO functional annotations responding to 5FU-induced myelosuppression. In comparison to the Control group, a total of 756 genes were upregulated in the Model group, while 1,147 genes were downregulated (**Figure 4A** and **Supplementary Table S2**, Control vs. Model). The GO analysis classified these 5FU-induced DEG into 64 biological process categories, with the top five functional annotation clusters being immune system processes, hematopoietic or lymphoid organ development, immune system development, hematopoiesis, and cell activation (**Figure 4B** and **Supplementary Table S3**).

The transcriptional profiles of the martynoside, R2, and Rg2 groups were analyzed. In comparison to the Model group, a total of 61 genes were upregulated and 120 were downregulated in the martynoside group (**Figure 4A** and **Supplementary Table S2**, Model vs. martynoside). These DEG were classified into 26 different functional categories, most of which belong to immune system processes, response to external stimuli, and regulation of multicellular organismal process groups. Although they were ranked quite low, biological categories that are directly related to hematopoietic function (i.e., hematopoietic or lymphoid organ development and homeostatic processes) were also identified (**Figure 4B** and **Supplementary Table S3**). In the R2 treatment group, a total of 826 upregulated and 660 downregulated genes were detected in comparison to the Model group (**Figure 4A**, **Supplementary Table S2**, Model vs. R2). These DEG were classified into 119 categories. The top functional annotation clusters were immune system processes, cell activation, leukocyte activation, hematopoietic or lymphoid organ development, and immune system development (**Figure 4B** and **Supplementary Table S3**). In the Rg2 treatment group, 509 upregulated genes and 233 downregulated genes, in comparison to the Model group, were observed (**Figure 4A** and **Supplementary Table S2**, Model vs. Rg2) and were classified into 66 categories. The top functional categories included all immune system processes, hematopoietic or lymphoid organ development, immune system development, hematopoiesis, and myeloid cell differentiation (**Figure 4B** and **Supplementary Table S3**).

We also compared the similarities and differences in the GO terms responsible for each treatment. As **Supplementary Table S3** shows, of the 26 GO terms enriched in the martynoside group, 22 (84.6%) were also observed to be enriched in the R2 group, while of the 66 GO terms enriched in the Rg2 group, 63 (95.4%) were also enriched in the R2 group. There were four GO term categories that were only enriched in the martynoside group: response to radiation, response to ionizing radiation, nucleosome organization, and protein-DNA complex subunit organization. There were also three unique categories enriched in the Rg2 group: B cell activation, response to metal ions, and response to lipoteichoic acid. Finally, there were 49 categories that were only enriched in the R2 group. These included leukocyte cell–cell adhesion, positive regulation of response to stimulus, and others.

We then analyzed the co-expressed DEG between the individual treatment groups vs. the Model group and the Model vs. Control groups. As shown in the Venn diagram (**Figure 4C**), there were 110 DEG in the martynoside group, 1,033 DEG in the R2 group, and 631 DEG in the Rg2 group that were related to those in the Model group. As shown in the heat map in **Figure 4D**, the variation trends (upward or downward) appeared to vary similarly between the Model vs. Control and Model vs. treatment groups. This indicates that martynoside, R2, and Rg2 can recover the expression of most co-expressed DEG changed by myelosuppression.

We also compared the DEG among the samples. As shown in the Venn diagram (**Figure 4C**), there were 60 co-regulated DEG following treatment with all three compounds, which included *Cxcl1*, *Cxcl2*, *Tnf*, *Acod1*, *Mc5r*, *Fosl1*, and others (**Supplementary Table S4**). The GO analysis classified these 60 genes into 56 biological process categories, including inflammatory response and chemokine-mediated signaling pathways (**Supplementary Table S5**, *p* < 0.05). The KEGG analysis showed that the DEG were significantly enriched in TNF signaling, cytokine–cytokine receptor interaction, toll-like receptor, and others (**Supplementary Table S6**, *p* < 0.05). As for the unique DEG, nine genes, including *Opn3* and *Supt4b*, were only regulated by martynoside; 440 genes, including *Egr4* and *Cdkn1a*, were only regulated by R2; and 85 genes, including *Tnn2* and *Car3*, were only regulated by Rg2. **Table 2** lists the top five genes regulated by only one of the three compounds (sorted by their absolute value of log2 (fold change)). It should be noted that we did not include predicted genes in this study (e.g., *Gm2775*).

**Table 2:** Top 5 DEG uniquely regulated by martynoside, R2, or Rg2 in comparison to the Model group.

Group	DEG	Fold change (log2,
		*vs.* Model)
Martynoside	*Rny1* (RNA, Y1 small cytoplasmic, Ro-associated)	-7.74
	*Hist1h4a* (histone cluster 1, H4a)	-1.85
	*Opn3* (RNA, Y1 small cytoplasmic, Ro-associated)	1.29
	*Supt4b* (suppressor of Ty 4B)	-1.21
	*Tnnt3* (troponin T3, skeletal, fast)	-1.15
R2	*Egr4* (early growth response 4)	-6.85
	*Cdkn1a* [cyclin-dependent kinase inhibitor 1A (P21)]	-3.33
	*Ets2* (E26 avian leukemia oncogene 2, 3′ domain)	-3.18
	*Sap25* (sin3 associated polypeptide)	2.98
	*Il4i1* (interleukin 4 induced 1)	2.93
Rg2	*Tnnc2* (troponin C2, fast)	2.75
	*Amigo2* (adhesion molecule with Ig like domain 2)	2.07
	*Col8a1* (collagen, type VIII, alpha 1)	-1.95
	*Map7* (microtubule-associated protein 7)	1.92
	*Car3* (carbonic anhydrase 3)	-1.70


### Verification of Compound-Responsive Genes by RT-qPCR

**FIGURE 5:**
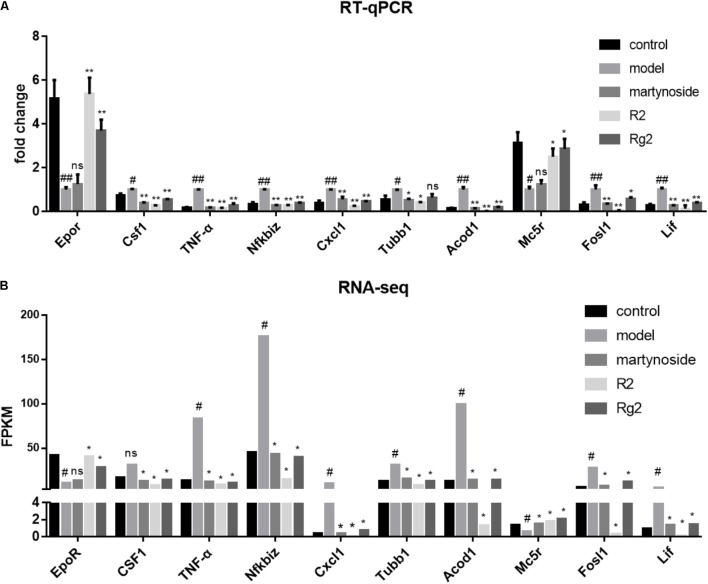
Verification of compound-responsive genes using RT-qPCR. **(A)** Total RNA was extracted from BMNCs in each treatment group. RT-qPCR data were expressed as fold change compared to Model. ^#^*p* < 0.05, ^##^*p* < 0.01, compared with the Control group; ^∗^*p* < 0.05, ^∗∗^*p* < 0.01, compared with the Model group; ns, no significant difference compared with the Model group. **(B)** Matched sample PFKM value from RNA-seq analysis. ^#^DEG compared with the Control group; ^∗^DEG compared with the Model group; ns, no significant difference.

We chose ten DEG (*Epor*, *Csf1*, and *TNF*-α, *Nfkbiz*, *Cxcl1*, *Tubb1*, *Acod1*, *Mc5r*, *fosl1*, and *Lif*) and validated their expression by real-time quantitative PCR (RT-qPCR) (**Figure 5A**). Meanwhile, the mean FPKM value for the same sample was listed (**Figure 5B**). The direction of fold change of all genes was consistent between RT-qPCR and RNA-seq. With the exceptions of *Mc5r*, *Tubb1*, and *Csf1*, the expression patterns of other seven genes were consistent between the RT-qPCR and RNA-seq analyses. In detail, for *Mc5r*, a significant difference between the martynoside group and the Model group was only observed in the RNA-seq data; for *Tubb1*, a significant difference between the Rg2 group and the Model group was only observed in the RNA-seq data; and for *Csf1*, a significant difference between the Model group and the control group was only observed in the RNA-seq data. The concordance rate of these two methods achieved 70%.

### Differential Expression of Hematopoiesis-Related Genes Following Martynoside, R2, or Rg2 Treatment

**FIGURE 6:**
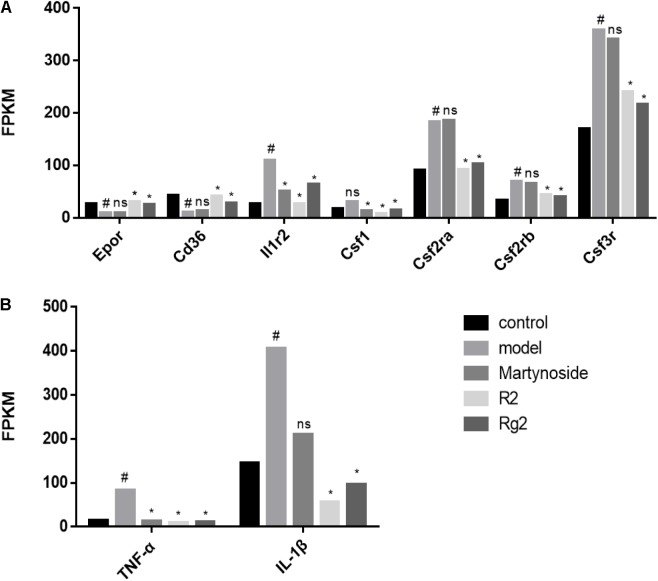
Hematopoiesis-related DEGs. **(A)** The expression/PFKM of colony formation-related genes. **(B)** The expression/PFKM of hematopoiesis-related genes. ^#^DEG compared with the Control group; ^∗^DEG compared with the Model group; ns, no significant difference.

To further validate the effects of these three compounds on hematopoietic function in the myelosuppression mouse model, we focused on the DEG related to colony formation or regulation of hematopoiesis. First, we analyzed the expression of cytokines related to colony formation. As **Figure 6A** shows, in comparison with the control group, the expression of *Epor* and *Cd36*, which are related to BFU-E cell function, was decreased, while the expression of *Csf3r*, *Csf2ra*, *Csf1*, and *Il1r2*, which are related to CFU-GM cell function, was increased during 5FU-induced myelosuppression. Treatment with martynoside, R2, or Rg2 resulted in increased *Epor* and *Cd36* expression and decreased expressions of the others. We also measured the expression of *IL-1*β and *TNF*-α, two hematopoiesis-related inflammatory genes. While 5FU myelosuppression significantly increased the expression of *IL-1*β and *TNF-*α, martynoside, R2, and Rg2 all individually decreased the expression of these genes, almost down to the same levels as the controls (**Figure 6B**). These results suggest that martynoside, R2, and Rg2 probably contribute to the maintenance of BM homeostasis.

### Hematopoiesis-Related Molecular Regulatory Networks for Martynoside, R2, or Rg2

**FIGURE 7:**
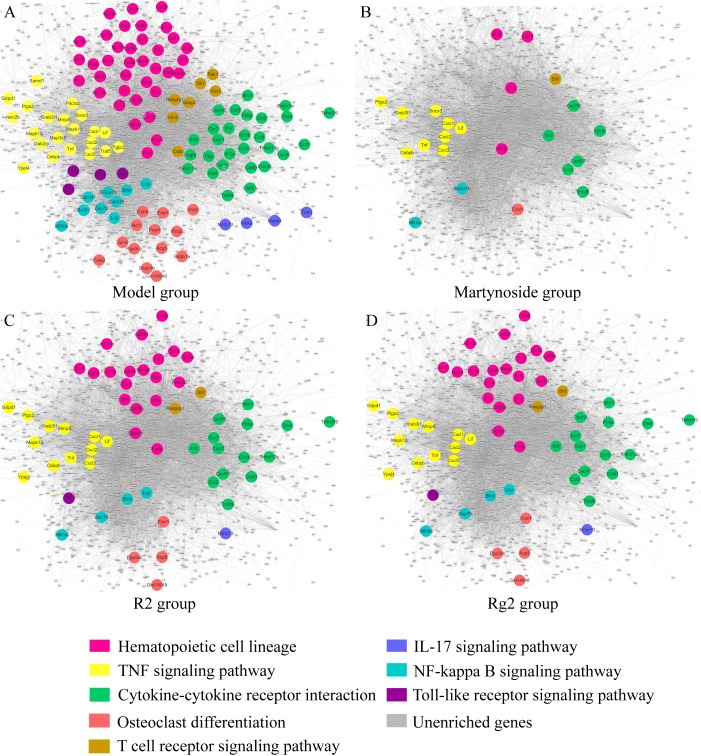
Hematopoiesis-related molecular regulatory networks for martynoside, R2, or Rg2. **(A)** Model group. **(B)** Martynoside group. **(C)** R2 group. **(D)** Rg2 group. The nodes are representative of DEG altered in the myelosuppression mouse model. The lines are representative of interactions between DEG. The color of node indicates enrichment in different pathways; gray nodes indicate genes that were not enriched in that treatment group.

Differentially expressed genes caused by myelosuppression were applied to KEGG pathway enrichment (**Supplementary Table S7**) and interaction network generation (**Figure 7**, the main network in gray). DEG related to hematopoietic functional pathways, including hematopoietic cell (HPC) lineage, TNF signaling pathways, osteoclast differentiation, cytokine–cytokine receptor interaction, T cell receptor signaling pathways, IL-17 signaling pathways, NF-κB signaling pathways, and toll-like receptor signaling pathways, were annotated with different colors and highlighted in the network (**Figure 7A**). As illustrated in **Figures 7B–D**, in which the co-regulated DEG due to individual treatment are highlighted, the expression of the relevant genes appears to be effectively regulated by individual treatment. All three compounds have the ability to regulate genes involved in HPC lineage, TNF signaling pathways, osteoclast differentiation, cytokine–cytokine receptor interaction, T cell receptor signaling pathways, and NF-κB signaling pathways. Notably, R2 and Rg2 appear to have very similar patterns with respect to pathway regulation (**Figures 7C,D**), and they also regulate genes involved in IL-17 signaling pathways and toll-like receptor signaling pathways.

## Discussion

As BM tissue is sensitive to chemotherapeutic drugs, high dosages of 5FU may cause severe BM suppression (Longley et al., 2003; Shaikh et al., 2016). 5FU-induced rapid BM damage is characterized by a marked decrease in the number of both total BM cells, which reach nadir at day 7, and peripheral blood cells, which reach nadir at day 10. Subsequently, if there is no further exposure, a post-damage repair phase occurs, in which BM and peripheral blood cell counts gradually increase, returning to normal levels around day 21 and after day 14, respectively (Zhang et al., 2009; Raghavendran et al., 2012; Kojouharov et al., 2014). In the current study, we utilized a mouse model for 5FU-induced myelosuppression (Wang et al., 2015) to evaluate the effects of three novel bioactive compounds found in FEJ. In addition to BMNCs counting, hematopoietic colony formation assay and FCM analysis of HSC (Lin^-^/c-kit^+^/Sca-1^+^) population, the gene expression profiles were investigated using RNA-seq. RNA-seq is an established approach to comprehensive and in-depth characterization of transcriptome profiling using deep-sequencing technologies (Li et al., 2016). In comparison to microarrays, RNA-seq quantifies more species, provides more precise transcript quantification, and covers a wider detection range (Ren et al., 2017). Using these techniques, we delineated the transcriptome maps of martynoside, Rg2, and R2 and characterized their therapeutic effects *in vivo.* The results provide valuable information concerning their underlying mechanisms during hematopoietic processes.

Bone marrow is a major hematopoietic organ, consist of HSC and BMSCs. The number of BMNCs is considered a direct reflection of BM health (Carey, 2003). In the present study, we found that the total number of BMNCs was significantly reduced in 5FU-treated mice on both day 10 and day 15 post-exposure, which indicates myelosuppression. Interestingly, treatment with martynoside for 10 days (**Figure 2A**), and treatment with Rg2 or R2 for 15 days (**Supplementary Figure S1**) increased BMNCs counts in comparison to that of the Model group, which suggests that treatment with these compounds increases BM health and could aid recovery after chemotherapy-induced myelosuppression.

We also evaluated the regeneration capacity of the BM after 5FU treatment using either a CFU assay to detect hematopoietic progenitor cells (HPCs). As a result from CFU assay, BFU-E and CFU-GM, which represent the proliferative capacity of erythroid progenitors and granulocyte/macrophage progenitor cells, respectively (Yang et al., 2011; Liu et al., 2014), were identified. While levels of both CFU-GM and BFU-E cells are decreased in the damage phase, increasing CFU-GM cell numbers reflect a repair phase in the BM (Yang et al., 2011; Liu et al., 2014). In our Model group, we observed significant decrease in numbers of both BFU-E and CFU-GM on day 10 post-exposure (**Figures 2C,D**), and a significant decrease in the number of BFU-E cells but an increase in the number of CFU-GM cells on day 15 post-exposure (**Supplementary Figure S1**), which are consistent with the patterns expected in the damage phase and repair phase, respectively. The treatment with martynoside, R2 or Rg2 for 10 days all resulted in stimulation of both BFU-E and CFU-GM numbers; the treatment with martynoside or Rg2 resulted in stimulation of BFU-E cell numbers. This suggests that martynoside, Rg2, and R2 could be used to accelerate BM restoration after myelosuppression and promote hematopoietic function.

In general, HPCs are derived from HSC and have the ability of proliferation and differentiation to generate all blood cell lineages to maintain system homeostasis (Broxmeyer et al., 2012; Hoggatt et al., 2013). HSC have the capacity to self-renew, proliferate, differentiate to various hematopoietic progenitors, and to generate specific blood cell types and hematopoietic cell lineage. Therefore, HSC are regarded as “seeds” in hematopoietic system (Warr et al., 2011). The cell surface markers of HSC in mice are recognized as Lin^-^/c-kit^+^/Sca-1^+^ (Jiang et al., 2016). Decreasing of the regenerative capacity and proliferation of HSC could be characterized in chemo- or radiotherapy injured BM (Kubota et al., 2009; Liu et al., 2014; Jiang et al., 2015). In the present study, HSC population was significantly on day10 days post-exposure. Treatment with martynoside, Rg2 or R2 increased HSC population in comparison to that of the Model group (**Figure 3**), which further suggests that treatment with three compounds protect BM from injury and accelerate hematopoietic cell lineage restoration after myelosuppression. Taken together, our results of BMNC counting, CFU assay and HSC confirmed that martynoside, R2 and Rg2 protected BM from 5FU-induced myelosuppression and enhanced hematopoiesis probably through directly promoting HPC and HSC.

To fully understand the beneficial effects of these FEJ components, it is necessary to evaluate their underlying mechanisms with respect to the biological pathways they regulate. In current study, the BM samples collected on day 15 post-exposure were sent for RNA-seq and bioinformatic analysis. The results of the GO and KEGG analyses performed indicate that martynoside, R2, and Rg2 probably regulate the HPC lineage pathway and influence the processes of hematopoiesis and hematopoietic or lymphoid organ development. It is well known that the HPC lineage pathway involves two categories of hematopoiesis-related cytokines: hematopoietic growth factors (HGFs) and hematopoietic inhibitory factors (HIFs). While HGFs, including *EPO*, *IL-1*β, *IL-6*, *SCF*, and *GM-CSF*, direct the division and maturation of hematopoietic stem/progenitor cells, HIFs, including *TGF-*β and *TNF-*α, inhibit these processes (Raghavendran et al., 2012; Liu et al., 2014). Our RNA-seq data and pathway analyses show that in the myelosuppression model, the expression of genes encoding hematopoiesis-related cytokines (i.e., *MCSF*, *IL-1*β, and *TNF-*α), as well as the hematopoiesis-related signaling pathways involved (i.e., the TNF-α signaling pathway), were altered. However, treatment with one of the three FEJ compounds adjusted these changes back to the control levels. One of the genes affected by R2 and Rg2 treatment was *EPOR*, which is a surface receptor for EPO cells that plays an important role in primary BM progenitors and in balancing red blood cell production (Ren et al., 2017). *Mc5R*, which is a melanocortin that is expressed ubiquitously in adults and embryos (Andersen et al., 2005), was greatly affected by all three treatments. During erythroblast differentiation, increasing expression of *Mc5R* plays a critical role in orthochromatic erythroblast enucleation (Simamura et al., 2015). Taken together, these findings suggest that martynoside, R2, and Rg2 all have the capacity to facilitate erythroid hematopoiesis via various mechanisms.

Notably, immune system processes are among the top GO biological processes identified in this study, and several DEG, including *Acod1*, *Cxcl1*, *Cxcl2*, *Nfkbiz*, *TLRs*, and other inflammatory factors were found to be altered during myelosuppression and subsequent treatment. The contribution of inflammation to aggravated damage of HSCs has been demonstrated for multiple inflammatory factors (Zhu et al., 2013; Zhou et al., 2014). Therefore, in addition to directly stimulating HSCs, it is likely that martynoside, R2, and Rg2 also inhibit inflammation in the hematopoietic microenvironment, thus facilitating recovery during chemotherapy-induced myelosuppression.

Although a number of co-regulated DEG and pathways were altered by all three compounds during myelosuppression, there were also genes that uniquely responded to martynoside, R2, or Rg2. The top-ranked DEG that was only upregulated by martynoside was *Opsin3* (*OPN3*). *OPN3*, also known as encephalopsin or panopsin, which is a member of the opsin family and is widely expressed in many tissues, especially in the eye, brain, and liver. Its ability to sense light for non-visual functions is well known, and it also acts as a G protein-coupled receptor in the activation of G protein-related downstream signaling cascades (Sugihara et al., 2016). Although no direct links between *OPN3* and 5FU treatment have been identified in normal cells, in hepatocellular carcinoma cells, *OPN3* is involved in regulating 5FU-induced apoptosis via control of the phospho-Akt/total Akt and Bcl2/Bax ratios (Jiao et al., 2012). As apoptosis in rapidly proliferating HPCs contributes to chemotherapy-induced acute myelosuppression (Li et al., 2011; Chen et al., 2017), we speculate that martynoside may protect the BM from 5FU-induced apoptosis via inhibition of *OPN3* expression.

Among the top-ranked downregulated DEG, cyclin-dependent kinase inhibitor 1A (*CDKN1A*) was one that responded only to R2 treatment. *CDKN1A*, or *p21*, plays an important role in modulating DNA repair processes, blocking cell cycle progression, and promoting apoptosis. It binds to and inhibits cyclin-dependent kinase activity, thereby preventing phosphorylation of critical cyclin-dependent kinase substrates (Esteve-Puig et al., 2014). In the present study, 5FU increased *CDKN1A* expression, which was reversed following R2 treatment. This suggests that R2 could inhibit HPC apoptosis and promote BM recovery during myelosuppression via a mechanism that differs from that of martynoside.

One of the top-ranked downregulated DEG that was only regulated by Rg2 was carbonic anhydrase 3 (*CAR 3*), which participates in the response to oxidative stress in erythrocytes. A previous study showed that increased *CAR3* expression promotes oxidative stress in BM (Kuo et al., 2005). In the present study, 5FU increased the expression of *CAR3* in the BM, which was reversed by Rg2 treatment. Oxidative stress is known to contribute to chemotherapy-induced myelosuppression in BM (Numazawa et al., 2011; Xiong et al., 2017), which leads us to believe that the observed Rg2-mediated improvement in hematopoietic function during myelosuppression may be modulated through decreased oxidative stress.

Fufang E’jiao Jiang is a TCM formula consisting of various components. Pharmacological and clinical studies have confirmed that FEJ has an exact therapeutic effect in the treatment of anemia and chemotherapy-induced BM suppression (Zhu et al., 2013; Zhou et al., 2014). Recently, by using radiotherapy and chemotherapy-induced myelosuppressed mice models, Liu et al. clearly confirmed that FEJ has the ability to promote the recovery of BM hematopoietic function (Liu et al., 2014). Interestingly, the bioactivity of FEJ was identified related to the improvement of BM hematopoietic microenvironment, to facilitate cell proliferation and prevent BMNCs from apoptosis, as well as to regulate the expressions of inflammation related cytokines including IL-6 and IL-1β, which is partially in accordance with our functional network analysis result. Our future work will be focus on validating biological functions of the three components at protein expression level as well as *in vivo*.

## Conclusion

In summary, we confirmed the hematopoietic effects of martynoside, Rg2, and R2, originally identified as bioactive ingredients of FEJ, in a 5FU-induced myelosuppression mouse model. These compounds stimulate BMNCs growth, as well as promote HSC and HPC cell expansion via mechanisms that involve the regulation of HPC lineage, NF-κB and TNF signaling, inhibition of inflammation, and acceleration of HPC recovery. Although these compounds share some mechanisms, they also function through their own unique mechanisms. By identifying the hematopoietic effects of and providing transcriptome maps for these compounds, the results of the present study provide an experimental basis for discovering the mechanisms by which FEJ and other traditional Chinese medicines act, in addition to advancing our understanding of radiotherapy- and chemotherapy-induced myelosuppression.

## Author Contributions

JQ generated the conception and designed the study. XL and ZH performed most of the experiments. XL, YZ, SG, WL, XZ, and YS analyzed and interpreted the data. HQ provided the technical support. JQ and XL drafted the article. JQ made the final approval of the version to be submitted.

## Conflict of Interest Statement

The authors declare that the research was conducted in the absence of any commercial or financial relationships that could be construed as a potential conflict of interest.

## Footnotes

1 www.genomics.org.cn

2 ftp://hgdownload.cse.ucsc.edu/goldenPath/mm10

3 http://www.ncbi.nlm.nih.gov/bioproject/407862

4 http://www.geneontology.org/

5 http://www.genome.jp/kegg/

6 https://david.ncifcrf.gov/home.jsp

7 http://bioinfogp.cnb.csic.es/tools/venny/

8 http://www.string-db.org/

## Supplementary Material

FIGURE S1 Effects of martynoside, R2, and Rg2 on hematopoietic function in 5-FU-induced myelosuppression mice. **(A)** The number of BMNCs from each group. Cells were isolated from each group and counted as described in Materials and methods. **(B)** Representative image of colony formation in each treatment group. CFU-GM: colony-forming units-granulocyte macrophage. BFU-E: burst forming unit-erythroid. **(C)** Number of BFU-E cells in each treatment group. **(D)** Number of CFU-GM cells in each treatment group. The results are expressed as the means ± SEM. (*n* = 3 for Control, *n* = 2 for other groups). ^##^*p* < 0.01, compared with the Control group; ^∗^*p* < 0.05, ^∗∗^*p* < 0.01, compared with the Model group. ns, no significant difference.

TABLE S1 Summary of the sequence assembly after RNA-seq.

TABLE S2 DEG in model group.

TABLE S3 GO analysis of model group.

TABLE S4 60 common genes between three compounds groups.

TABLE S5 GO analysis of 60 common genes.

TABLE S6 KEGG analysis of 60 common genes.

TABLE S7 KEGG analysis of model group.
