# Supplementary figures and images for: Transcriptome Profiling Analysis Reveals the Potential Mechanisms of Three Bioactive Ingredients of Fufang E’jiao Jiang During Chemotherapy-Induced Myelosuppression in Mice
Source: Front Pharmacol. 2018 Jun 13;9:616. doi: 10.3389/fphar.2018.00616 (PMC6008481; doi:10.3389/fphar.2018.00616)

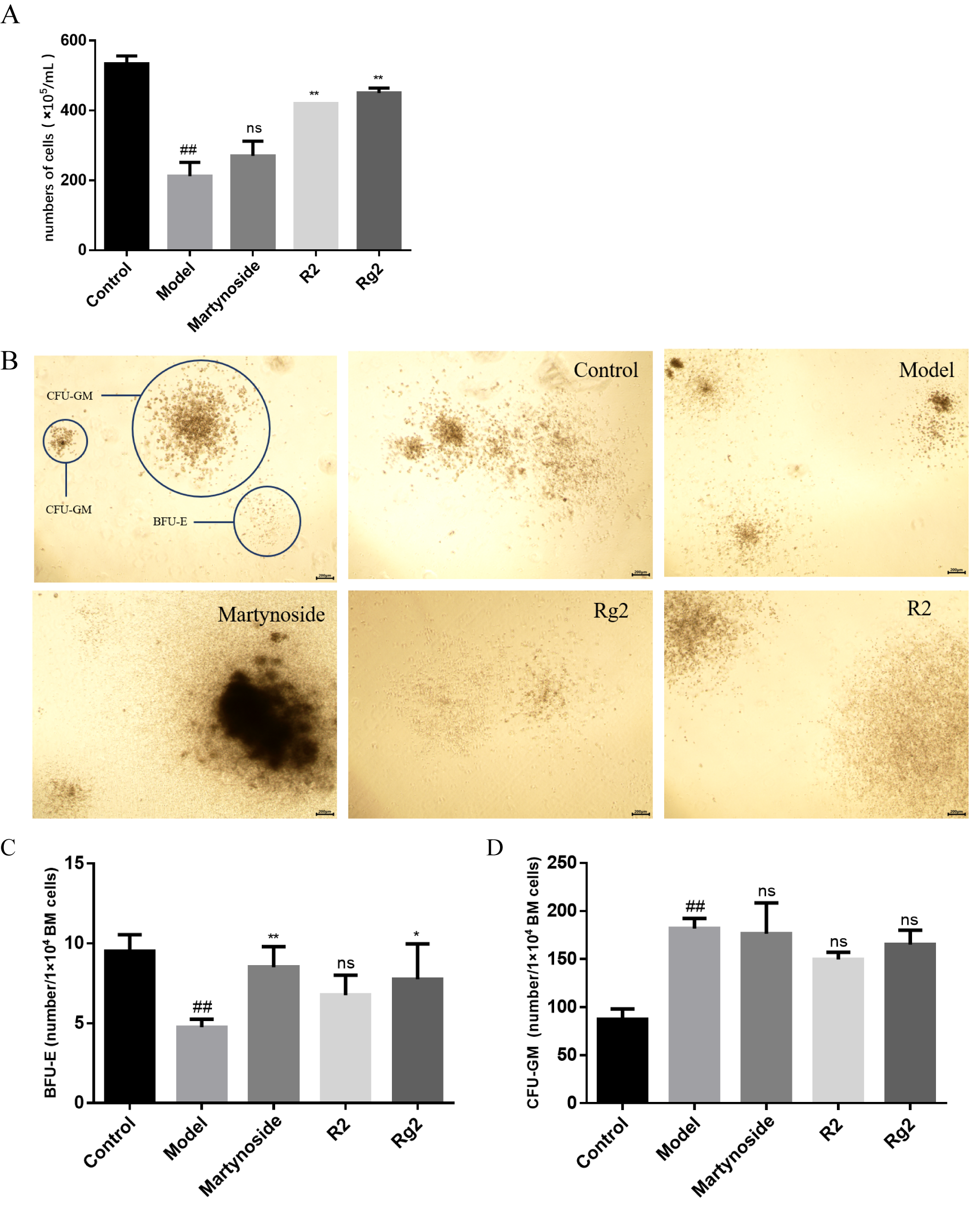

Supplement: FIGURE S1 — Effects of martynoside, R2, and Rg2 on hematopoietic function in 5-FU-induced myelosuppression mice. (A) The number of BMNCs from each group. Cells were isolated from each group and counted as described in Materials and methods. (B) Representative image of colony formation in each treatment group. CFU-GM: colony-forming units-granulocyte macrophage. BFU-E: burst forming unit-erythroid. (C) Number of BFU-E cells in each treatment group. (D) Number of CFU-GM cells in each treatment group. The results are expressed as the means ± SEM. (n = 3 for Control, n = 2 for other groups). ##p < 0.01, compared with the Control group; ∗p < 0.05, ∗∗p < 0.01, compared with the Model group. ns, no significant difference. [file Image_1.TIF]
