# Supplementary material for: Transcriptome Profiling Analysis Reveals the Potential Mechanisms of Three Bioactive Ingredients of Fufang E’jiao Jiang During Chemotherapy-Induced Myelosuppression in Mice
Source: Front Pharmacol. 2018 Jun 13;9:616. doi: 10.3389/fphar.2018.00616 (PMC6008481; doi:10.3389/fphar.2018.00616)
Supplement: TABLE S1 — Summary of the sequence assembly after RNA-seq. [file Table_1.docx]

Table S1. Summary of the sequence assembly after RNA-seq

| Sample | Raw Data Size (bp) | Raw Reads Number | Clean Data Size (bp) | Clean Reads Number | Total Mapped Reads (%) | Unique Match (%) | Clean Read Q20 (%) ≥90 |
| --- | --- | --- | --- | --- | --- | --- | --- |
| Control_1 | 1,206,857,400 | 24,137,148 | 1,200,708,500 | 24,014,170 | 99.49 | 69.19 | 97.6 |
| Control_2 | 1,206,861,550 | 24,137,231 | 1,201,848,800 | 24,036,976 | 99.58 | 66.89 | 98.3 |
| Control_3 | 1,206,855,050 | 24,137,101 | 1,201,814,000 | 24,036,280 | 99.58 | 64.64 | 98 |
| Model_1 | 1,206,853,850 | 24,137,077 | 1,201,583,500 | 24,031,670 | 99.56 | 74.81 | 98.1 |
| Model_2 | 1,206,854,750 | 24,137,095 | 1,201,757,550 | 24,035,151 | 99.57 | 73.77 | 98.2 |
| Martynoside_1 | 1,206,858,050 | 24,137,161 | 1,199,293,950 | 23,985,879 | 99.37 | 75.06 | 98.1 |
| Martynoside_2 | 1,206,862,300 | 24,137,246 | 1,194,864,950 | 23,897,299 | 99 | 73.67 | 98.1 |
| R2_1 | 1,206,854,450 | 24,137,089 | 1,202,742,250 | 24,054,845 | 99.65 | 63.53 | 98.3 |
| R2_2 | 1,206,863,000 | 24,137,260 | 1,204,181,150 | 24,083,623 | 99.77 | 67.73 | 98.2 |
| Rg2_1 | 1,206,860,150 | 24,137,203 | 1,204,699,700 | 24,093,994 | 99.82 | 68.4 | 98.1 |
| Rg2_2 | 1,206,862,500 | 24,137,250 | 1,203,843,750 | 24,076,875 | 99.74 | 66.39 | 98.3 |
