# Supplementary material for: Transcriptome Profiling Analysis Reveals the Potential Mechanisms of Three Bioactive Ingredients of Fufang E’jiao Jiang During Chemotherapy-Induced Myelosuppression in Mice
Source: Front Pharmacol. 2018 Jun 13;9:616. doi: 10.3389/fphar.2018.00616 (PMC6008481; doi:10.3389/fphar.2018.00616)
Supplement: TABLE S4 — 60 common genes between three compounds groups. [file Table_4.DOCX]

Table S4. 60 common genes between three compounds groups

| Gene ID | Gene name |
| --- | --- |
| 14825 | Cxcl1 |
| 20310 | Cxcl2 |
| 64818 | Krt81 |
| 384244 | Gm5294 |
| 319150 | Hist1h3b |
| 16365 | Acod1 |
| 16153 | Il10 |
| 16175 | Il1a |
| 21926 | Tnf |
| 16878 | Lif |
| 53311 | Mybph |
| 211472 | Olfr1373 |
| 19225 | Ptgs2 |
| 442829 | Ccin |
| 12475 | Cd14 |
| 80885 | Hcar2 |
| 14283 | Fosl1 |
| 15945 | Cxcl10 |
| 14066 | F3 |
| 80859 | Nfkbiz |
| 100503468 | Gm14023 |
| 20613 | Snai1 |
| 66102 | Cxcl16 |
| 54611 | Pde3a |
| 26427 | Creb3l1 |
| 21930 | Tnfaip6 |
| 54199 | Ccrl2 |
| 21950 | Tnfsf9 |
| 12608 | Cebpb |
| 330122 | Cxcl3 |
| 436062 | Fam92b |
| 243743 | Plxna4 |
| 320292 | Rasgef1b |
| 21664 | Phlda1 |
| 12142 | Prdm1 |
| 69573 | Hilpda |
| 225058 | Gm4832 |
| 18035 | Nfkbia |
| 20302 | Ccl3 |
| 72536 | Tagap |
| 545486 | Tubb1 |
| 100504112 | Ccer2 |
| 54698 | Crtam |
| 69319 | 1700001K23Rik |
| 22695 | Zfp36 |
| 17873 | Gadd45b |
| 17203 | Mc5r |
| 71446 | Wrb |
| 20400 | Sh2d1a |
| 625360 | BC147527 |
| 223267 | Ggact |
| 108900 | Fam72a |
| 67900 | Mtfp1 |
| 69885 | Aunip |
| 71041 | Pcgf6 |
| 435684 | Shf |
| 15505 | Hsph1 |
| 75572 | Acyp2 |
| 545384 | BC094916 |
| 15511 | Hspa1b |
