# Supplementary material for: Transcriptome Profiling Analysis Reveals the Potential Mechanisms of Three Bioactive Ingredients of Fufang E’jiao Jiang During Chemotherapy-Induced Myelosuppression in Mice
Source: Front Pharmacol. 2018 Jun 13;9:616. doi: 10.3389/fphar.2018.00616 (PMC6008481; doi:10.3389/fphar.2018.00616)
Supplement: TABLE S5 — GO analysis of 60 common genes. [file Table_5.DOCX]

Table S5. GO analysis of 60 common genes.

| Term | PValue | Genes |
| --- | --- | --- |
| GO:0006954~inflammatory response | 1.51E-10 | 12475, 15945, 14825, 21926, 330122, 20310, 16365, 80859, 16175, 54199, 16153, 20302, 19225 |
| GO:0071222~cellular response to lipopolysaccharide | 6.19E-09 | 20310, 330122, 16365, 12475, 22695, 15945, 16153, 12608, 21926, 66102 |
| GO:0006955~immune response | 6.05E-08 | 21950, 20310, 330122, 16878, 15945, 16175, 16153, 14825, 20302, 21926 |
| GO:0032496~response to lipopolysaccharide | 7.34E-08 | 20310, 330122, 16365, 18035, 15945, 12608, 14825, 19225, 21926 |
| GO:0050715~positive regulation of cytokine secretion | 2.45E-06 | 12475, 16175, 16153, 54698, 21926 |
| GO:0042127~regulation of cell proliferation | 3.24E-06 | 21950, 20310, 330122, 18035, 12142, 15945, 19225, 21926 |
| GO:0002237~response to molecule of bacterial origin | 5.93E-06 | 20310, 12475, 16153, 14825 |
| GO:0070098~chemokine-mediated signaling pathway | 1.72E-05 | 20310, 330122, 15945, 14825, 20302 |
| GO:0006935~chemotaxis | 2.01E-05 | 20310, 330122, 54199, 15945, 20302, 66102 |
| GO:0031663~lipopolysaccharide-mediated signaling pathway | 8.99E-05 | 12475, 18035, 20302, 21926 |
| GO:0010818~T cell chemotaxis | 1.16E-04 | 15945, 20302, 66102 |
| GO:0030593~neutrophil chemotaxis | 9.71E-04 | 20310, 330122, 14825, 20302 |
| GO:0060707~trophoblast giant cell differentiation | 0.001159349 | 16878, 12142, 20613 |
| GO:0071407~cellular response to organic cyclic compound | 0.001237107 | 18035, 12608, 20302, 21926 |
| GO:0051384~response to glucocorticoid | 0.001237107 | 20310, 16153, 19225, 21926 |
| GO:0060326~cell chemotaxis | 0.001385514 | 20310, 15945, 14825, 20302 |
| GO:0071347~cellular response to interleukin-1 | 0.001490405 | 20310, 16365, 12608, 20302 |
| GO:0045944~positive regulation of transcription from RNA polymerase II promoter | 0.001680363 | 15505, 16878, 18035, 26427, 16175, 14283, 16153, 12608, 20302, 21926 |
| GO:0050728~negative regulation of inflammatory response | 0.001896332 | 16365, 22695, 21930, 16153 |
| GO:0034612~response to tumor necrosis factor | 0.002244738 | 12475, 19225, 66102 |
| GO:0043065~positive regulation of apoptotic process | 0.00247597 | 17873, 108900, 14283, 19225, 21664, 21926 |
| GO:0030335~positive regulation of cell migration | 0.002551828 | 20613, 21930, 15945, 20302, 66102 |
| GO:0090023~positive regulation of neutrophil chemotaxis | 0.002813134 | 20310, 330122, 14825 |
| GO:0097421~liver regeneration | 0.003016082 | 18035, 16153, 12608 |
| GO:0008285~negative regulation of cell proliferation | 0.004436042 | 16878, 16175, 14283, 16153, 19225, 21926 |
| GO:0051930~regulation of sensory perception of pain | 0.00596161 | 16175, 16153, 20302 |
| GO:0007204~positive regulation of cytosolic calcium ion concentration | 0.008397878 | 20310, 330122, 14825, 20302 |
| GO:0032800~receptor biosynthetic process | 0.008438076 | 16153, 21926 |
| GO:0032755~positive regulation of interleukin-6 production | 0.009452035 | 21950, 16175, 21926 |
| GO:0032729~positive regulation of interferon-gamma production | 0.009452035 | 21950, 12475, 21926 |
| GO:0009408~response to heat | 0.01127525 | 20310, 12475, 15511 |
| GO:0006898~receptor-mediated endocytosis | 0.012437562 | 15505, 12475, 66102 |
| GO:0007566~embryo implantation | 0.012437562 | 16365, 16878, 19225 |
| GO:0002740~negative regulation of cytokine secretion involved in immune response | 0.014024638 | 16153, 21926 |
| GO:0042493~response to drug | 0.015187429 | 14283, 16153, 19225, 54611, 20302 |
| GO:0000165~MAPK cascade | 0.016221993 | 22695, 20302, 21926 |
| GO:0046330~positive regulation of JNK cascade | 0.016669321 | 17873, 16175, 21926 |
| GO:0031622~positive regulation of fever generation | 0.019580338 | 19225, 21926 |
| GO:0006915~apoptotic process | 0.021817497 | 17873, 80885, 435684, 67900, 21664, 21926 |
| GO:0034097~response to cytokine | 0.021929361 | 14283, 19225, 66102 |
| GO:0000185~activation of MAPKKK activity | 0.022346667 | 17873, 21926 |
| GO:0002376~immune system process | 0.022662642 | 20400, 16365, 12475, 12142, 54698 |
| GO:0010628~positive regulation of gene expression | 0.025852686 | 16878, 12142, 16175, 20302, 21926 |
| GO:1990440~positive regulation of transcription from RNA polymerase II promoter in response to endoplasmic reticulum stress | 0.030599821 | 26427, 12608 |
| GO:0034142~toll-like receptor 4 signaling pathway | 0.030599821 | 12475, 18035 |
| GO:0030728~ovulation | 0.030599821 | 21930, 19225 |
| GO:0007267~cell-cell signaling | 0.034190412 | 21950, 20400, 20302 |
| GO:0051091~positive regulation of sequence-specific DNA binding transcription factor activity | 0.036650221 | 14283, 16153, 21926 |
| GO:0071356~cellular response to tumor necrosis factor | 0.038538763 | 16365, 22695, 20302 |
| GO:0001775~cell activation | 0.038784659 | 20302, 21926 |
| GO:0001649~osteoblast differentiation | 0.040463934 | 26427, 20613, 20302 |
| GO:0033280~response to vitamin D | 0.044203538 | 15945, 19225 |
| GO:0050995~negative regulation of lipid catabolic process | 0.044203538 | 80885, 21926 |
| GO:0045670~regulation of osteoclast differentiation | 0.046901734 | 12608, 21926 |
| GO:0030889~negative regulation of B cell proliferation | 0.049592463 | 12142, 16153 |

p<0.05
