# Supplementary material for: Transcriptome Profiling Analysis Reveals the Potential Mechanisms of Three Bioactive Ingredients of Fufang E’jiao Jiang During Chemotherapy-Induced Myelosuppression in Mice
Source: Front Pharmacol. 2018 Jun 13;9:616. doi: 10.3389/fphar.2018.00616 (PMC6008481; doi:10.3389/fphar.2018.00616)
Supplement: TABLE S6 — KEGG analysis of 60 common genes. [file Table_6.DOCX]

Table S6. KEGG analysis of 60 common genes.

| Term | PValue | Genes |
| --- | --- | --- |
| mmu04668:TNF signaling pathway | 4.64E-10 | 20310, 330122, 16878, 18035, 26427, 15945, 12608, 14825, 19225, 21926 |
| mmu05134:Legionellosis | 1.17E-07 | 20310, 330122, 12475, 18035, 15511, 14825, 21926 |
| mmu05132:Salmonella infection | 1.76E-05 | 20310, 330122, 12475, 16175, 14825, 20302 |
| mmu04060:Cytokine-cytokine receptor interaction | 6.27E-05 | 21950, 16878, 15945, 16175, 16153, 20302, 21926, 66102 |
| mmu05140:Leishmaniasis | 1.47E-04 | 18035, 16175, 16153, 19225, 21926 |
| mmu04062:Chemokine signaling pathway | 1.55E-04 | 20310, 330122, 18035, 15945, 14825, 20302, 66102 |
| mmu04620:Toll-like receptor signaling pathway | 8.44E-04 | 12475, 18035, 15945, 20302, 21926 |
| mmu05133:Pertussis | 0.003753 | 12475, 16175, 16153, 21926 |
| mmu05164:Influenza A | 0.005761 | 18035, 15511, 15945, 16175, 21926 |
| mmu05152:Tuberculosis | 0.006375 | 12475, 16175, 16153, 12608, 21926 |
| mmu04064:NF-kappa B signaling pathway | 0.007985 | 12475, 18035, 19225, 21926 |
| mmu05142:Chagas disease (American trypanosomiasis) | 0.009414 | 18035, 16153, 20302, 21926 |
| mmu05145:Toxoplasmosis | 0.012108 | 18035, 15511, 16153, 21926 |
| mmu04380:Osteoclast differentiation | 0.016213 | 18035, 16175, 14283, 21926 |
| mmu05162:Measles | 0.019844 | 20400, 18035, 15511, 16175 |
| mmu04010:MAPK signaling pathway | 0.021851 | 17873, 12475, 15511, 16175, 21926 |
| mmu05321:Inflammatory bowel disease (IBD) | 0.02605 | 16175, 16153, 21926 |
| mmu05202:Transcriptional misregulation in cancer | 0.033239 | 12475, 80859, 12608, 319150 |
| mmu04622:RIG-I-like receptor signaling pathway | 0.033869 | 18035, 15945, 21926 |
| mmu05323:Rheumatoid arthritis | 0.047589 | 16175, 20302, 21926 |
| mmu04640:Hematopoietic cell lineage | 0.049691 | 12475, 16175, 21926 |

p<0.05
